# BAP1 loss induces mitotic defects in mesothelioma cells through BRCA1-dependent and independent mechanisms

**DOI:** 10.1038/s41388-022-02577-3

**Published:** 2022-12-22

**Authors:** Anita Singh, Sara Busacca, Aarti Gaba, Michael Sheaff, Charlotte Poile, Apostolos Nakas, Joanna Dzialo, Aleksandra Bzura, Alan G. Dawson, Dean A. Fennell, Andrew M. Fry

**Affiliations:** 1 Department of Molecular and Cell Biology, University of Leicester, Lancaster Road, Leicester, LE1 9HN UK; 2 Leicester Cancer Research Centre, Department of Genetics and Genome Biology, University of Leicester, Robert Kilpatrick Clinical Sciences Building, Leicester, LE2 7LX UK; 3 Department of Histopathology, Barts Health NHS Trust, Queen Mary University of London, The Royal London Hospital, London, E1 2ES UK; 4 University Hospitals of Leicester NHS Trust, Glenfield Hospital, Leicester, LE3 9QP UK

**Keywords:** Mitosis, Mesothelioma

## Abstract

The tumour suppressor BRCA1-associated protein 1 (BAP1) is the most frequently mutated cancer gene in mesothelioma. Here we report novel functions for BAP1 in mitotic progression highlighting the relationship between BAP1 and control of genome stability in mesothelioma cells with therapeutic implications. Depletion of BAP1 protein induced proteasome-mediated degradation of BRCA1 in mesothelioma cells while loss of BAP1 correlated with BRCA1 loss in mesothelioma patient tumour samples. BAP1 loss also led to mitotic defects that phenocopied the loss of BRCA1 including spindle assembly checkpoint failure, centrosome amplification and chromosome segregation errors. However, loss of BAP1 also led to additional mitotic changes that were not observed upon BRCA1 loss, including an increase in spindle length and enhanced growth of astral microtubules. Intriguingly, these consequences could be explained by loss of expression of the KIF18A and KIF18B kinesin motors that occurred upon depletion of BAP1 but not BRCA1, as spindle and astral microtubule defects were rescued by re-expression of KIF18A and KIF18B, respectively. We therefore propose that BAP1 inactivation causes mitotic defects through BRCA1-dependent and independent mechanisms revealing novel routes by which mesothelioma cells lacking BAP1 may acquire genome instability and exhibit altered responses to microtubule-targeted agents.

## Introduction

Malignant mesothelioma is a lethal cancer caused by exposure to asbestos with few treatment options [1]. Although immune checkpoint blockade has demonstrated an improved survival in phase III trials [2, 3], options in the relapsed setting are limited and molecularly stratified therapies are lacking. The microtubule poison vinorelbine that leads to microtubule depolymerization and activation of the spindle assembly checkpoint (SAC) in mitosis has demonstrated some useful clinical activity in the relapsed setting but is not licenced [4].

Human BAP1 is a 729 amino acid predominantly nuclear deubiquitinase (DUB), encoded on chromosome 3p21.1, and its inactivation occurs in upwards of 65% of mesotheliomas primarily through somatic mutation and copy number loss [5–9]. Inactivation of BAP1 leads to either loss of expression or cytoplasmic accumulation, which is commonly evaluated by immunohistochemistry at diagnosis [8, 10, 11]. BAP1’s deubiquitinase activity is encoded by the N-terminal 240 residues encoding an ubiquitin carboxyl hydrolase (UCH) domain. The C-terminal half of BAP1 was initially reported to associate directly with the RING domain of BRCA1, while the region between residues 182–356 binds to BARD1. However, BRCA1 and BARD1 also interact directly to form an E3 ubiquitin ligase [12]. Hence, while BAP1 and BRCA1-BARD1 mediate opposing deubiquitinating and ubiquitinating activities, respectively, it remains to be determined how exactly BAP1 regulates BRCA1 [6, 13–15].

In a similar manner to p53, the importance of BAP1 as a tumour suppressor resides in the breadth of its cellular functions, from regulating DNA transcription and repair in the nucleus to control of calcium flux in the cytoplasm [6, 16, 17]. This latter activity results from BAP1-mediated deubiquitylation of IP_3_ receptors on the endoplasmic reticulum (ER) that leads to calcium moving from the ER to mitochondria promoting cell death [18]. BAP1 may have additional cytoplasmic roles in regulating metabolism as loss of BAP1 is associated with the increase in aerobic glycolysis and reduced mitochondrial respiration typical of the Warburg effect [19].

Here, we were particularly interested in potential roles of BAP1 in controlling mitotic progression and chromosome segregation and hence how loss of BAP1 contributes to genome instability. Nuclear BAP1 plays a major role in regulating gene expression through assembly into several transcriptional complexes [20–27]. Indeed, BAP1 regulates the cell cycle through E2F1 responsive promoters in an HCF-1 dependent manner, and E2F1 controls expression of SAC regulators, including MAD2L1 and SIL [28]. BRCA1 can ubiquitinate γ-tubulin and this has been reported to inhibit microtubule nucleation and prevent centrosome duplication, although the mechanisms remain unclear [29–31]. BAP1 can deubiquitinate γ-tubulin [32], as well as microspherule protein 1 (MCRS1), a protein that caps the minus ends of mitotic microtubules to prevent their depolymerization [33, 34]. More recently, BAP1 was shown to interact with and stabilise via de-ubiquitination DIDO1, a centrosome-associated protein that co-localizes with γ-tubulin and whose disruption leads to centrosome amplification and chromosomal instability [35, 36]. Both BAP1 and BRCA1 are also involved in the DNA damage response and loss of either gene abrogates DNA repair and can act as a biomarker for targeted therapies with PARP inhibitors [1].

Based on this evidence, we hypothesized that BAP1 is an important regulator of mitotic events and that its loss could promote genome instability through defects in these processes. Furthermore, BAP1 status in mesothelioma patients might be an important predictor of response to microtubule poisons that interfere with mitotic spindle assembly, as well as mediators of the DNA damage response. However, it was unclear to what extent these might depend on its interaction with BRCA1. We therefore set out to characterise the consequences of BAP1 loss on mitotic progression in mesothelioma cells and discovered that BAP1 loss induces mitotic defects through both BRCA1-dependent and independent mechanisms.

## Results

### BAP1 protein is required for BRCA1 expression in mesothelioma cells and tumours

**Fig. 1:**
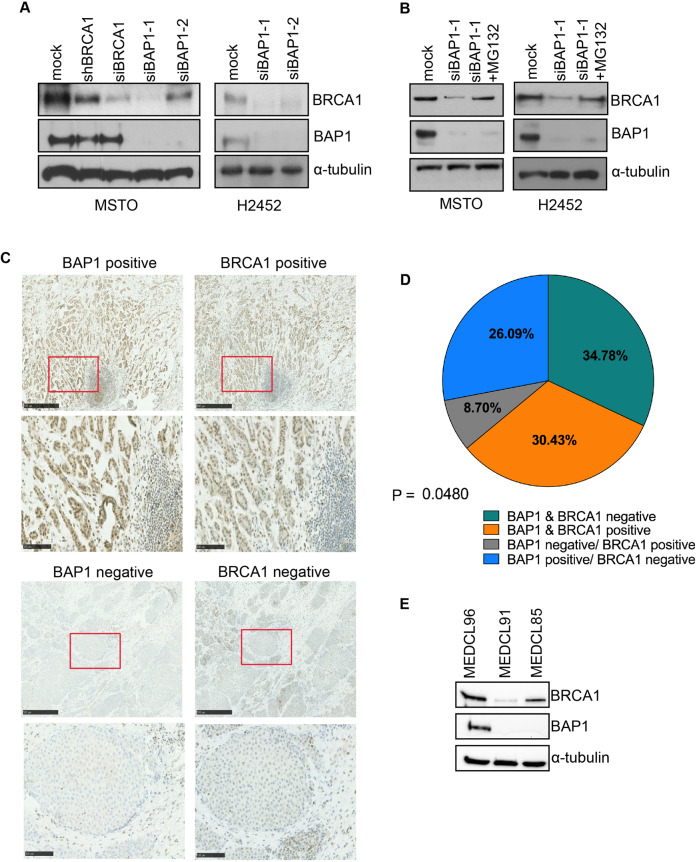
BAP1 regulates BRCA1 protein expression in cell lines and patient tumours. **A** MSTO-211H and NCI-H2452 cells were either mock-depleted or depleted with siRNAs against BRCA1 or BAP1 for 72 h, or by induction of an shBRCA1 sequence for 48 h. Cell lysates were analysed by Western blot with antibodies against BRCA1, BAP1 and α-tubulin. **B** MSTO-211H and NCI-H2452 cells were either mock-depleted or depleted with siRNAs against BAP1 for 72 h before being either untreated or treated with 20 µM MG132 for 6 h as indicated. Cell lysates were analysed by Western blot with antibodies against BRCA1, BAP1 and α-tubulin. **C** Representative immunohistochemical staining of BAP1 (left panels) and BRCA1 (right panels) on malignant mesothelioma patient tissues from the MEDUSA cohort (*n* = 26). Representative images from samples scored as positive or negative are shown at low (upper panels) and high (lower panels) magnification Scale bars are 500 µm in top panels and 100 µm in lower panels. **D** Pie chart showing the percentage of human mesothelioma tumours that stained negative or positive for protein expression of BAP1 and BRCA1. The p value indicates the significance of correlation for expression. **E** Cell extracts prepared from the primary human mesothelioma cell lines indicated were analysed by Western blot with antibodies against BRCA1, BAP1 and α-tubulin.

BRCA1 forms a complex with BARD1 to generate an active E3 ubiquitin ligase that can auto-ubiquitinate to promote its own destruction, while BAP1 is a DUB that by deubiquitinating BARD1 can disrupt this complex and reduce its turnover [12, 15]. Before setting out to explore how loss of BAP1 might regulate mitotic events in mesothelioma, we wished to examine the interdependence of BAP1 and BRCA1 protein expression in mesothelioma cells by Western blot. For this purpose, we used MSTO-211H cells that have wild-type BAP1 and NCI-H2452 cells that have an A95D mutation in the UCH domain of BAP1 that renders it catalytically inactive as a DUB [37, 38]. Using two independent siRNAs, we found that BAP1 depletion led to a significant reduction in BRCA1 protein in the MSTO-211H and NCI-H2452 mesothelioma cell lines (Fig. 1A). However, there was no change in BAP1 protein expression following either siRNA-mediated depletion of BRCA1 or inducible expression of an shRNA against BRCA1 in MSTO-211H cells (Fig. 1A).

To determine whether this loss of BRCA1 in BAP1-depleted cells was a consequence of altered protein stability, we examined BRCA1 protein expression following BAP1 depletion in the presence of the proteasome inhibitor, MG132. Following depletion of BAP1 for 72 h, treatment with MG132 for 6 h led to a substantial rescue of BRCA1 expression in MSTO-211H and NCI-H2452 cells indicating that BAP1 inhibits the proteasome-mediated degradation of BRCA1 (Fig. 1B). Interestingly, as BAP1 is catalytically inactive as a DUB in NCI-H2452 cells, the loss of BRCA1 from these cells upon BAP1 depletion and rescue by MG132 argues that BAP1 controls the ubiquitylation state of BRCA1 in a manner that is independent of its DUB activity.

We next examined whether the frequent loss of BAP1 in mesothelioma tumours in patients correlates with loss of BRCA1 protein expression. Previous immunohistochemical analyses for BAP1 protein have demonstrated that patient-associated deletions, insertions or point mutations all lead to loss of nuclear staining, even though cytoplasmic staining is present in some patients [8, 39], Here, we evaluated the expression of BAP1 and BRCA1 protein in tumour samples taken from chemonaive mesothelioma patients undergoing extended pleurectomy decortication (*n* = 26) using immunohistochemistry (Fig. 1C). We identified a positive and significant correlation between BAP1 and BRCA1 protein expression (Chi-square test for association, *p* < 0.05; Fig. 1D).

We then analysed BAP1 and BRCA1 protein expression in three primary mesothelioma cell lines (Fig. 1E). MEDCL96 is wild-type for BAP1, whereas MEDCL85 has a mutation in the HBM binding domain (*R417fs)* that regulates interaction with host cell factor-1 (HCF-1). MEDCL91 has a frame-shift mutation in the BRCA1 binding domain (*E631fs*). None of these primary cell lines have a BRCA1 mutation. The two cell lines with BAP1 mutations showed no protein expression of BAP1 and had low or no protein expression of BRCA1 compared to MEDCL96 cells where BAP1 and BRCA1 were clearly detected. Collectively, these data are consistent with the hypothesis that loss of BAP1 may impact BRCA1 expression in vivo.

### BAP1 and BRCA1 are required for accurate mitotic progression in mesothelioma cells

**Fig. 2:**
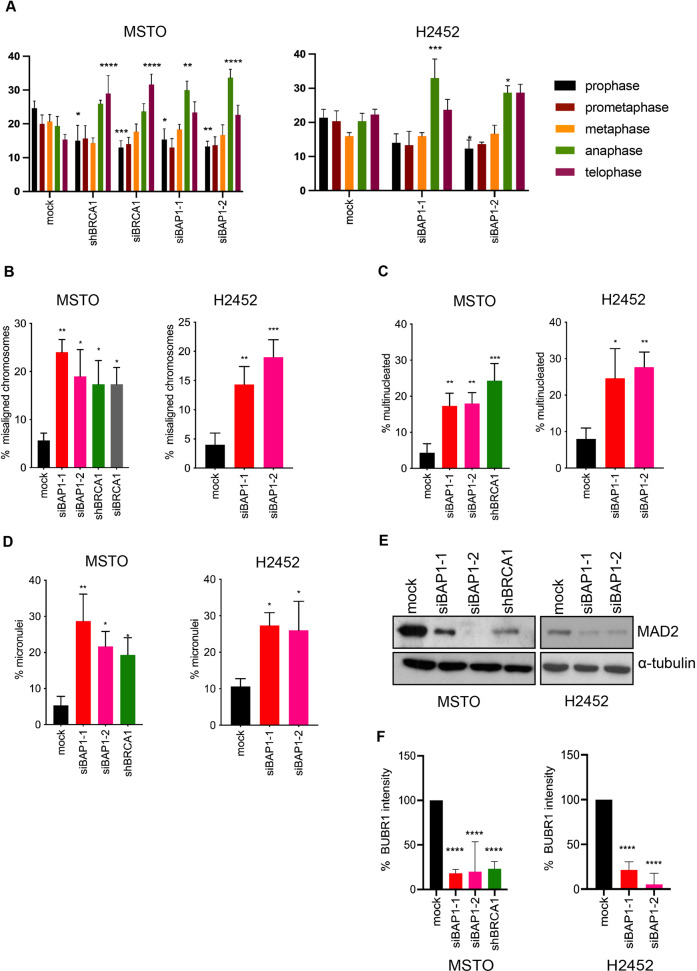
BAP1 depletion leads to mitotic progression defects and loss of SAC integrity. **A** MSTO-211H and NCI-H2452 were either mock-depleted or depleted of BAP1 or BRCA1 as described in Fig. 1A. Cells were fixed and stained with Hoechst 33258 to observe the DNA and histograms show the percentage of mitotic cells in the different mitotic phases indicated. **B** Cells treated and stained as in A, were analysed to measure the percentage of metaphase cells with misaligned chromosomes (means *±* S.D.) based on *n* = 3 independent experiments (60 mitotic cells for each condition). **C**, **D** The percentage of interphase cells treated as in A that were multinucleated (**C**) or contained micronuclei (**D**) were scored. **E** Cell lysates treated as in A were analysed by Western blot with antibodies against of MAD2L1 and α-tubulin. **F** Cells treated as in A were processed for immunofluorescence microscopy with BUBR1 antibodies and DNA stained with Hoechst 33258. Histograms show the mean intensity of BUBR1 on metaphase chromosomes following BAP1 or BRCA1 depletion relative to that observed upon mock depletion. Data in **A**, **B**, **C**, **D** and **F** are expressed as means *±* S.D. (*n* = 3).

To begin to examine the impact of BAP1 loss on mitotic progression, we first used flow cytometry to assess cell cycle distribution following BAP1 or BRCA1 depletion. Although some studies have reported that depletion of BAP1 or BRCA1 inhibits S-phase progression [15, 40], we observed no significant difference by flow cytometry in cell cycle distribution or percentage of cells in G2/M upon BAP1 or BRCA1 depletion in MSTO-211H or NCI-H2452 cells (Fig. S1A, B). These results are consistent with no observed change in cell cycle profile in skin fibroblasts taken from BAP1 heterozygous individuals that had reduced BAP1 protein levels [18], although different responses in other cell types cannot be excluded. We then performed fluorescence microscopy on cells fixed and stained for DNA to analyse the relative proportion of cells in different stages of mitosis. This revealed that depletion of either BAP1 or BRCA1 resulted in an increase in the proportion of mitotic cells in late mitosis, i.e. anaphase and telophase, compared to mock-depleted cells (Fig. 2A). However, while BRCA1 led to most mitotic cells being present in telophase, BAP1 depletion led to the highest proportion of mitotic cells being in anaphase. This suggests that while BAP1 and BRCA1 are both important for normal mitotic progression of mesothelioma cells, they are likely to have at least in part distinct functions.

Further analysis of these mitotic cells by immunofluorescence microscopy revealed that depletion of either BAP1 or BRCA1 led to chromosome congression errors with an increase in the frequency of misaligned chromosomes in metaphase (Fig. 2B, and Fig. S2A). The frequency of misaligned chromosomes was similar whether cells were depleted of BAP1 or BRCA1. Depletion of either BAP1 or BRCA1 also led to a substantial increase in the number of interphase cells that contained multiple nuclei or micronuclei (Fig. 2C, D, and Fig. S2B). Staining with the centromere marker, CENP-A, revealed the presence of centromeres within these micronuclei indicating that they were likely the result of chromosome segregation errors rather than fragments of broken chromosomes arising due to DNA repair defects [41, 42].

The increased proportion of mitotic cells in late mitosis and the presence of indicators of chromosome segregation errors in interphase cells is consistent with cells slipping through the SAC before full chromosome congression. Indeed, loss of SAC integrity is known to lead to chromosome segregation errors during unperturbed mitotic progression, as well as during drug-induced mitotic arrest [43, 44]. Since previous data had shown that BRCA1 controls SAC activity through regulating expression of MAD2L1, a key SAC component [45], we examined the protein levels of MAD2L1 in cells depleted of BAP1. In comparison to mock-depleted cells, there was a marked reduction in MAD2L1 protein upon depletion of either BAP1 or BRCA1 in MSTO-211H cells, or depletion of BAP1 in NCI-H2452 cells (Fig. 2E). We then analysed the expression and localization of another SAC component, BUBR1, in mitotic cells. Although there was no difference in expression of BUBR1 protein as determined by Western blotting upon BAP1 or BRCA1 depletion in MSTO-211H cells (Fig. S2C), there was reduced association with kinetochores upon depletion of either BAP1 or BRCA1 in MSTO-211H and NCI-H2542 cells arrested in prometaphase with vinorelbine (Fig. 2F, and Fig. S2D). These data indicate that BAP1 is likely to regulate the SAC in a similar manner to BRCA1 through control of MAD2L1 expression and recruitment of BUBR1 to kinetochores.

### BAP1 and BRCA1 are required to maintain spindle bipolarity and normal centrosome number

**Fig. 3:**
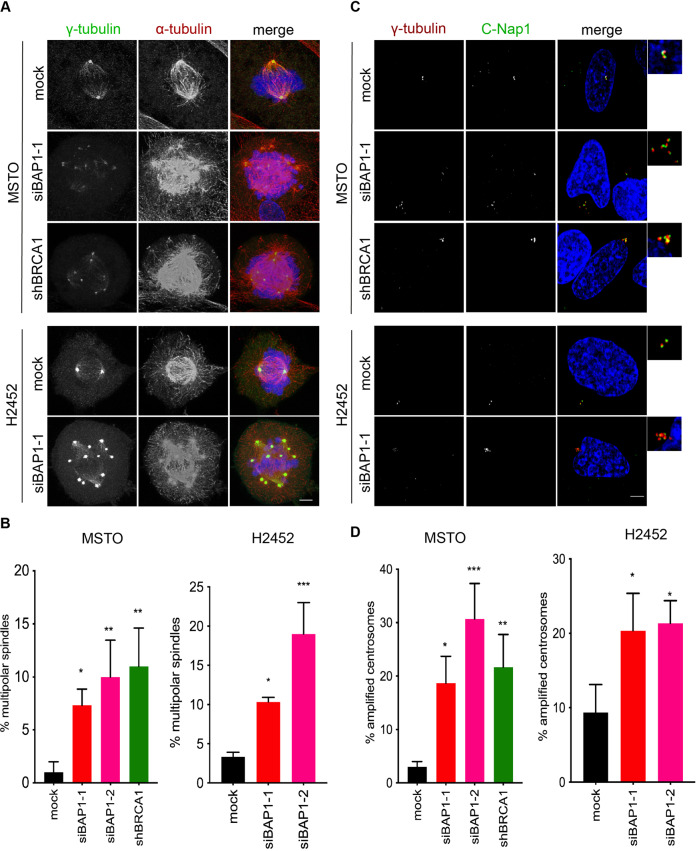
BAP1 or BRCA1 loss induces multipolar spindles and centrosome amplification. **A** MSTO-211H and NCI-H2452 cells were either mock-depleted or depleted of BAP1 or BRCA1 as described in Fig. 1A before analysis by immunofluorescence microscopy with antibodies against γ-tubulin (green) and α-tubulin (red). Merge images include DNA stained with Hoechst 33258 (blue). **B** Histogram shows the percentage of cells with multipolar spindles. **C** MSTO-211H:shBRCA1 and NCI-H2452 cells were either mock-depleted or depleted of BAP1 or BRCA1 as described in Fig. 1A before analysis by immunofluorescence microscopy with antibodies against γ-tubulin (red) and C-Nap1 (green). Merge images include DNA stained with Hoechst 33258 (blue). **D** Histograms show the percentage of mitotic cells with amplified centrosomes. Scale bars in **A** and **C**, 6 μm.

The errors observed in chromosome congression in metaphase cells depleted of BAP1 or BRCA1 suggest defects in spindle organization or chromosome attachment. Examination of mitotic cells by immunofluorescence microscopy with antibodies against α-tubulin and γ-tubulin revealed an increased frequency of multipolar spindles following either BAP1 or BRCA1 depletion in MSTO-211H and NCI-H2452 cells (Fig. 3A, B). The frequency of multipolar spindles in MSTO-211H cells was similar following BAP1 or BRCA1 depletion suggesting that BAP1 regulation of spindle organization may be at least in part through control of BRCA1 expression.

As multipolar spindles may result from the presence of amplified centrosomes, we next examined centrosome number in interphase cells. For this purpose, immunofluorescence microscopy analysis was performed with antibodies against γ-tubulin, a marker of pericentriolar material, and C-Nap1, a marker of centrioles. Cells were scored as possessing amplified centrosomes if they had > 2 centrosomes. This revealed that depletion of either BAP1 or BRCA1 led to a similar, approximate ten-fold, increase in the percentage of cells with amplified centrosomes in MSTO-211H cells (Fig. 3C, D). These results are consistent with previous studies demonstrating a role for BRCA1 in preventing centrosome amplification [31, 46]. Depletion of BAP1 in NCI-H2452 cells also led to an increase in the proportion of cells with amplified centrosomes albeit with a lower fold-increase; however, parental NCI-H2452 cells have a higher basal level of centrosome amplification than MSTO-211H cells. The lower frequency of multipolar spindles in mitosis compared to amplified centrosomes in interphase in response to BAP1 or BRCA1 depletion may be explained by the ability of cancer cells to cluster amplified centrosomes to form pseudo-bipolar spindles. Nonetheless, amplified centrosomes are associated with increased levels of chromosome congression and segregation defects due to an increased frequency of merotelic kinetochore attachments [47, 48].

### BAP1 loss leads to reduced centrosome size in interphase and mitosis

**Fig. 4:**
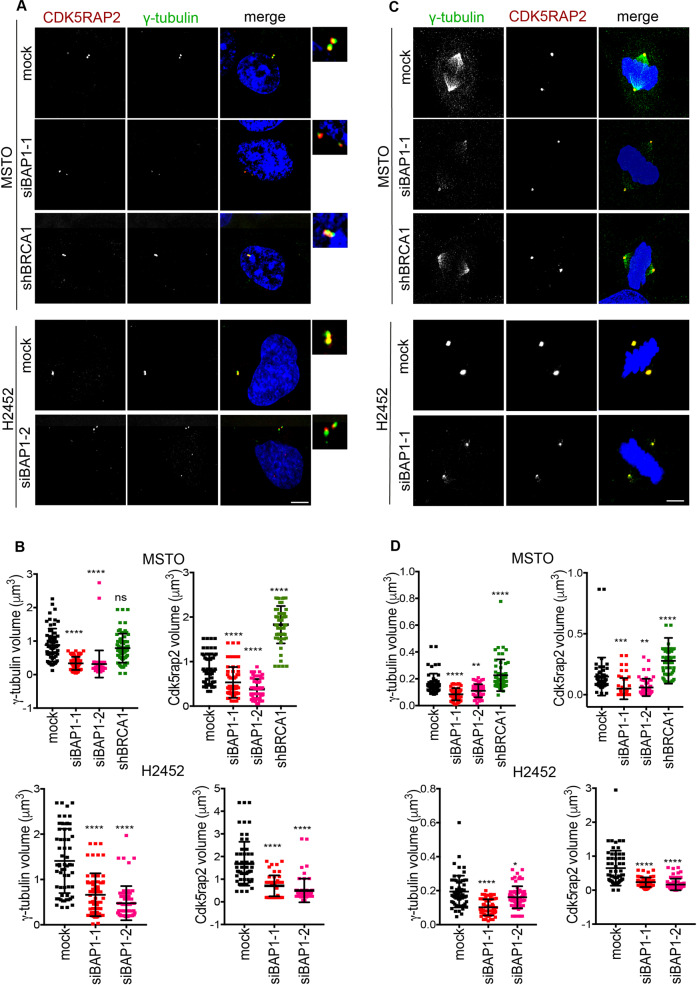
Loss of BAP1 but not BRCA1 causes reduced centrosome volume. **A** MSTO-211H and NCI-H2452 cells were either mock-depleted or depleted of BAP1 or BRCA1 as described in Fig. 1A before analysis by immunofluorescence microscopy with antibodies against γ-tubulin (green) and CDK5RAP2 (red). Merge images include DNA (blue) stained with Hoechst 33258. Magnified views of centrosomes are shown. **B** Dot plots show the γ-tubulin and CDK5RAP2 volume in mock and siBAP1 depletion in MSTO-211H and NCI-H2452 interphase cells taken from **A**. **C** Cells treated as in **A** were stained with γ-tubulin (green) and CDK5RAP2 (red) antibodies. **D** Dot plots represent the γ-tubulin and CDK5RAP2 volume in metaphase cells taken from **C**. Volume measurements were performed using Imaris 3D on *n* = 30 cells. Scale bars in **A** and **C**, 5 μm.

How BAP1 or BRCA1 controls centrosome number remains unclear. It is plausible that amplified centrosomes arise as a result of cells exiting mitosis without completing cytokinesis, in a similar manner to how multinucleated cells can be generated. Indeed, the percentage of interphase cells that were multinucleated and have amplified centrosomes upon BAP1 or BRCA1 depletion was similar. However, in complex with BARD1, BRCA1 has been reported to mono-ubiquitinate γ-tubulin, which in turn is required for centrosome duplication raising the possibility that BRCA1 controls centrosome number via a more direct mechanism involving γ-tubulin modification [31, 49, 50]. Indeed, BRCA1 localises to centrosomes throughout the cell cycle and BAP1 co-localizes with γ-tubulin at centrosomes in mitosis [32, 51–53]. Furthermore, BRCA1 loss has been associated with accumulation of γ-tubulin at centrosomes [54, 55]. To examine whether BAP1 loss leads to a similar increase in γ-tubulin at centrosomes, we performed immunofluorescence microscopy using antibodies against γ-tubulin, as well as CDK5RAP2, another component of the centrosome involved in microtubule nucleation [56, 57]. Consistent with these previous results, depletion of BRCA1 led to an increase in centrosome size in interphase MSTO-211H and NCI-H2452 cells as measured not only with γ-tubulin but also CDK5RAP2. However, in contrast, depletion of BAP1 led to a significant decrease in centrosome size during interphase as determined with both centrosome markers (Fig. 4A, B).

At the onset of mitosis, centrosome maturation leads to an increase in centrosome size and enhanced microtubule nucleation. This occurs through recruitment of additional γ-tubulin and CDK5RAP2, as well as other centrosome proteins. We therefore analysed centrosome size in mitotic cells depleted of BRCA1 and BAP1 by immunofluorescence microscopy with antibodies against γ-tubulin and CDK5RAP2. Following BRCA1 depletion, there was no discernible change in γ-tubulin content in mitotic MSTO-211H cells as compared to mock-depleted mitotic cells; however, there was an unexpected increase in CDK5RAP2 upon BRCA1 depletion in mitotic MSTO-211H cells (Fig. 4C, D). In contrast, BAP1 depletion led to a significant reduction in the volume of both γ-tubulin and CDK5RAP2 at centrosomes in mitotic MSTO-211H and NCI-H2452 cells (Fig. 4C, D). These data reveal a striking difference in the response of cells to depletion of BRCA1 or BAP1. In turn, this raises the prospect that loss of BAP1 could have additional consequences on mitotic progression through mechanisms above and beyond the control of BRCA1 expression.

### BAP1 but not BRCA1 regulates spindle length and spindle pole attachment

**Fig. 5:**
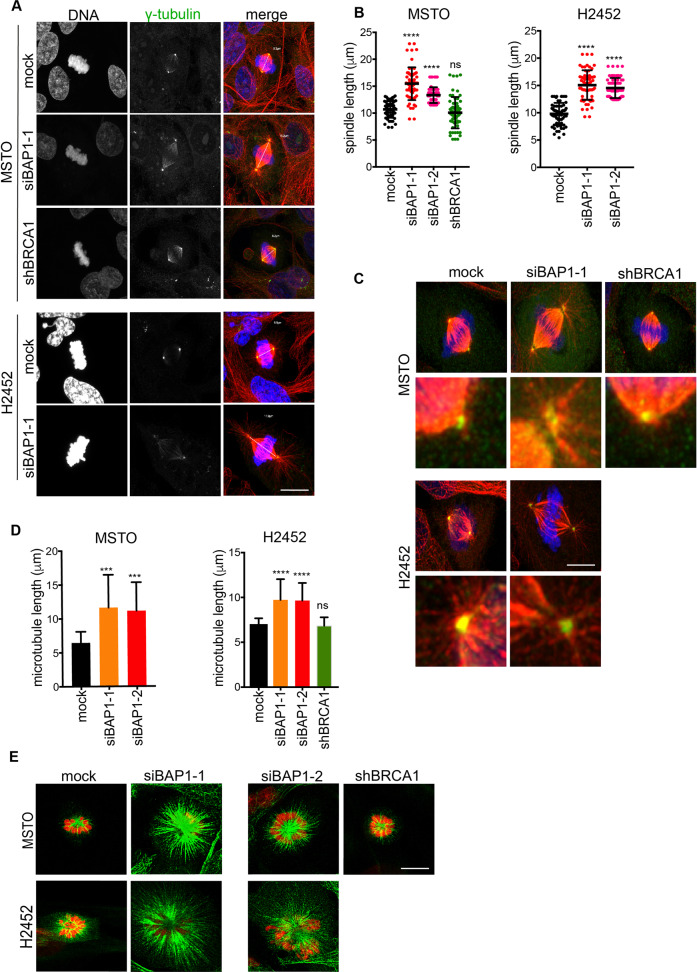
Loss of BAP1 but not BRCA1 causes increased spindle microtubule length. **A** MSTO-211H and NCI-H2452 cells were either mock-depleted or depleted of BAP1 or BRCA1 as described in Fig. 1A before analysis by immunofluorescence microscopy with antibodies against γ-tubulin (green) and α-tubulin (red); DNA (blue) is stained with Hoechst 33258. Merge images include lines drawn from pole-to-pole from which measurements of spindle length were made (yellow). **B** Dot plots show the spindle length based on images shown in **A**. Quantification of spindle lengths is taken from three independent experiments. For each condition, 30 mitotic cells were scored. **C** Detachment of poles from spindle microtubules was observed in some cells depleted of BAP1, but not in mock- or BRCA1-depleted cells. Cells were stained as in **A**. Magnified images (right hand panels). **D** Cells depleted of BAP1 or BRCA1 as in **A** were then incubated with STLC for 6 h to generate monopolar spindles. Cells were stained with α-tubulin antibodies (green); DNA is stained with Hoechst 33258 (red). Histogram shows the length of microtubules quantified from the centre of the monopolar aster to the microtubule tips. **E** Representative images of cells treated and stained as in **D**. Scale bars in **A**, **C** and **E**, 8 μm.

Further examination of mitotic cells by immunofluorescence microscopy with γ-tubulin and α-tubulin antibodies revealed that bipolar spindles were less round and more elliptical in shape following depletion of BAP1 as compared to mock- or BRCA1-depleted cells (Fig. 5A). Determination of spindle length by measuring pole-to-pole distance revealed that spindles were on average 20–50% longer following depletion of BAP1 in MSTO-211H cells (depending on the siRNA sequence used) and 50% longer following depletion of BAP1 in NCI-H2452 cells (Fig. 5B). In contrast, depletion of BRCA1 in MSTO-211H cells did not affect spindle length (Fig. 5A, B). Intriguingly, following BAP1 depletion, a small number of mitotic MSTO-211H and NCI-H2452 cells were observed to have spindle poles that were clearly detached from the bulk of the spindle microtubules (Fig. 5C). This defect was never observed in mock-depleted or BRCA1-depleted cells. Astral microtubules also appeared more abundant and longer in the BAP1-depleted MSTO-211H and NCI-H2452 cells than in mock-depleted or BRCA1-depleted cells (see Fig. 5A, C). As an alternative approach to quantify the length of microtubules in mitosis, depleted cells were treated with STLC, an Eg5 inhibitor, to arrest cells in prometaphase with a monopolar spindle. Measurement of microtubule length in the monopolar spindle asters revealed a ~20% and ~80% increase in the length of microtubules upon BAP1 depletion in MSTO-211H and NCI-H2452 cells, respectively, but no change upon depletion of BRCA1 in MSTO-211H cells (Fig. 5D, E). Taken together, these results suggest that BAP1 contributes to regulation of microtubule length and spindle pole attachment during mitosis in mesothelioma cells in a manner that is independent of BRCA1.

### BAP1 controls microtubule length in mitosis through regulating KIF18 expression

**Fig. 6:**
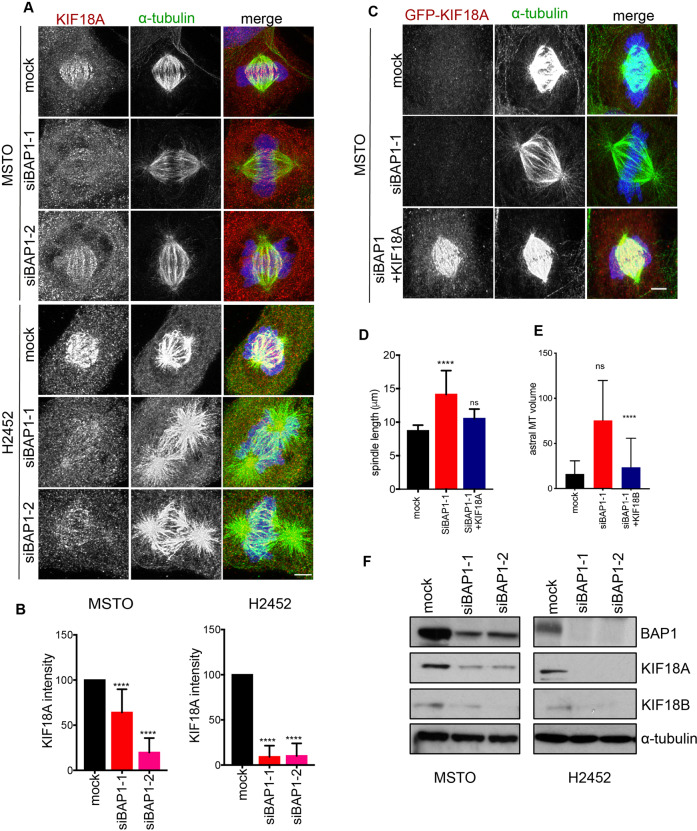
BAP1 depletion leads to loss of KIF18A and KIF18B. **A** MSTO-211H and NCI-H2452 cells were either mock-depleted or depleted of BAP1 or BRCA1 as described in Fig. 1A before analysis by immunofluorescence microscopy with antibodies against α-tubulin (green) and KIF18A (red). Merge images include DNA (blue) stained with Hoechst 33258. **B** Histograms show KIF18A intensity at kinetochores relative to mock-depleted cells (100%) quantified from images shown in **A**. **C** MSTO-211H cells were mock-depleted or depleted of BAP1 for 48 h prior to transfection of GFP-KIF18A for 24 h as indicated. Cells were fixed and stained for GFP (red) and α-tubulin (green). Merge images include DNA (blue) stained with Hoechst 33258. **D** Histogram shows the spindle length as quantified from images shown in **C**. **E** MSTO-211H cells were mock-depleted or depleted of BAP1 for 48 h prior to transfection of GFP-KIF18B for 24 h. Astral microtubule (MT) volumes were determined in the area from the pole towards the cell cortex away from the main spindle using Imaris 3D. **F** MSTO-211H and NCI-H2452 cells were either mock-depleted or depleted with the siRNAs indicated against BAP1 for 72 h before extracts were prepared and analysed by Western blot with antibodies against BAP1, KIF18A, KIF18B and α-tubulin. Scale bars in **A** and **C**, 5 μm.

The temporal and spatial control of spindle microtubule organization is critical to accurate chromosome segregation in mitosis [58]. Interestingly, the alterations in spindle shape and microtubule length observed in response to BAP1 depletion were highly reminiscent of phenotypes previously described to result from depletion of the mitotic kinesins, KIF18A and KIF18B, that possess microtubule destabilization activity [59]. KIF18A localizes to the plus-ends of kinetochore microtubules to control spindle length and chromosome positioning, while KIF18B plays a more specific role in regulating astral microtubule length [60–62]. To determine whether the elongated spindles observed after BAP1 depletion are due to perturbation of KIF18 proteins, we first analysed the localization of KIF18A on spindle microtubules by immunofluorescence microscopy in MSTO-211H and NCI-H2452 cells. This revealed substantial loss of KIF18A from kinetochore microtubules after BAP1 depletion (Fig. 6A, B). Strikingly, overexpression of GFP-KIF18A, which localised to kinetochore microtubules, in BAP1-depleted MSTO-211H cells restored spindle length to that seen in mock-depleted cells (Fig. 6C, D). BRCA1 depletion was not examined in this regard as no spindle length alteration was observed.

Previous work had shown that KIF18B, but not KIF18A, regulates the length of astral microtubules [63, 64]. In this case, attempts to detect endogenous KIF18B by immunofluorescence microscopy with several different commercial antibodies proved unsuccessful. Strikingly though, the ~5-fold increase in astral microtubule volume resulting from BAP1 depletion was reduced in cells transfected with GFP-KIF18B to the level observed in mock-depleted cells (Fig. 6E, and Fig. S3A). In contrast, the increased spindle length seen upon depletion of BAP1 was not rescued by overexpression of KIF18B (Fig. S3B). As these defects in spindle and astral microtubule length mediated by BAP1 depletion could be rescued by overexpression of KIF18A and KIF18B, respectively, we examined the expression of these kinesins in BAP1-depleted cells by Western blot. This revealed a significant reduction in both KIF18A and KIF18B proteins in MSTO-211H and NCI-H2452 cells following BAP1 depletion as compared to mock-depleted cells (Fig. 6F). We also found by Western blot that expression of KIF18A was increased in NCI-H226 cells stably expressing wild-type BAP1 compared to the parental cells that are null for BAP1 (Fig. S3C). KIF18B is mutant in NCI-H226 cells and so was not analysed [65]. Taken together, these data provide persuasive evidence that BAP1 regulates mitotic spindle organization through control of KIF18A and KIF18B protein expression.

## Discussion

BAP1 and BRCA1 are encoded by tumour suppressor genes that are frequently lost in human cancers, with *BAP1* the most commonly mutated gene in mesothelioma. The proteins are known to functionally interact though how they regulate each other and whether their loss has equivalent consequences are incompletely understood. Our data revealed that depletion of BAP1 leads to loss of BRCA1 protein expression in mesothelioma-derived cell lines but not vice versa. Moreover, loss of expression of BAP1 and BRCA1 proteins is strongly correlated in tumour samples taken from mesothelioma patients. Cellular BRCA1 protein levels are tightly regulated by transcriptional and post-translational mechanisms. Indeed, previous global mRNA expression profiling suggests that BAP1 depletion can perturb BRCA1 mRNA levels [27]. BAP1 forms a ternary complex with HCF-1 and Yin Yang 1 (YY1), a zinc finger multifunctional protein that can repress and activate many genes. Indeed, YY1 positively regulates BRCA1 expression by binding to its promoter [66]. BRCA1 can also be transcriptionally repressed through methylation of its promoter [67]. However, we found that BAP1 is likely to control BRCA1 expression at least in part at the level of protein stability in mesothelioma cells, as its loss upon BAP1 depletion was blocked by proteasome inhibition. This did not though appear to depend on the DUB activity of BAP1 as BRCA1 loss was also observed in response to BAP1 depletion in NCI-H2452 cells that express a catalytically inactive version of BAP1 [14, 15]. A number of E3 ubiquitin ligases have been identified, including HERC2, SCF^FBX044^ and HUWE1, that are capable of BRCA1 ubiquitination and future studies will be required to test whether they are involved in BAP1-dependent regulation of BRCA1 protein stability [67].

**Fig. 7:**
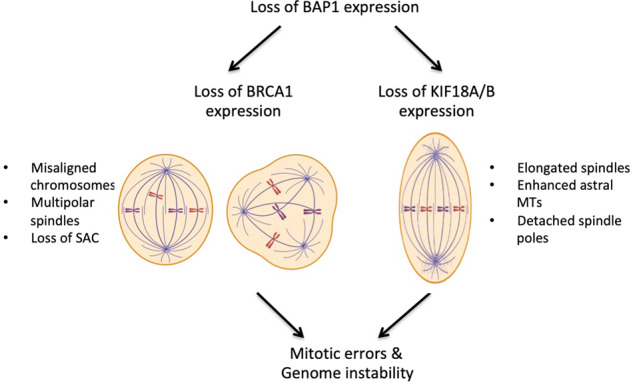
BRCA1-dependent and independent mitotic functions of BAP1. The schematic model illustrates how loss of BAP1 promotes mitotic errors and genome instability through BRCA1-dependent and -independent mechanisms. Loss of BAP1 expression is shown to lead to loss of BRCA1 on the left and loss of KIF18A and KIF18B on the right. Mitotic phenotypes associated with each pathway are indicated.

Microscopic analysis revealed an increased proportion of mitotic cells that had been depleted of BAP1 or BRCA1 in late mitosis. This is suggestive of a weakened SAC that is normally required to maintain timely progression through mitosis even in the absence of microtubule poisons. Loss of SAC integrity would be expected to lead to chromosome segregation errors and indeed cells with both multiple nuclei and micronuclei were observed upon BAP1 or BRCA1 depletion. We had previously reported that BRCA1 contributes to SAC integrity in mesothelioma cells in the presence of the microtubule poison, vinorelbine, through regulating the expression and localization of the SAC components, MAD2L1 and BUBR1, respectively [68]. Here, we demonstrated similar roles for BAP1 in controlling MAD2L1 expression and BUBR1 localization to kinetochores in non-drug treated mesothelioma cells. In addition, we observed centrosome amplification and multipolar spindle formation in BAP1 and BRCA1-depleted cells. These defects can arise as a result of failure of previous rounds of cell division. Hence, BAP1 and BRCA1 are both essential for accurate mitotic progression of mesothelioma cells with the role of BAP1 likely explained in part by its regulation of BRCA1 expression. Importantly though, we observed a number of extra mitotic phenotypes upon depletion of BAP1 that did not occur upon BRCA1 depletion indicating the presence of additional mechanisms through which BAP1 may contribute to mitotic progression and genome stability (Fig. 7).

First, we observed reduced centrosome size in interphase and mitosis upon BAP1 depletion as measured by the two pericentriolar material markers, γ-tubulin and CDK5RAP2. Moreover, in a small proportion of BAP1-depleted mitotic cells with bipolar spindles, the spindle poles were visibly detached from the main body of the spindle. BAP1 has previously been shown to co-localise with γ-tubulin at spindle poles in mitosis and, given the role of BAP1 in γ-tubulin ubiquitination and the fact that γ-tubulin interacts with CDK5RAP2, it is possible that depletion of BAP1 disturbs centrosome organization due to alterations in modification of γ-tubulin, or possibly DIDO1, that are distinct from those that occur upon BRCA1 loss [32, 56, 57]. Second, we found that BAP1 has a BRCA1-independent role in controlling the length of spindle and astral microtubules in mitosis through promoting expression of KIF18A and KIF18B, members of the kinesin-8 family of microtubule-based motors. Both these proteins bind to the plus-ends of microtubules and promote microtubule depolymerization [69]. As well as leading to elongated spindles, presumably as a result of failure of spindle microtubule depolymerization, KIF18A depletion leads to excessive chromosome oscillations, misalignment and segregation errors [59, 61, 70–72]. Meanwhile, KIF18B has a specific role in regulating the length, and potentially number, of astral microtubules.

Importantly, rescue experiments demonstrated that overexpression of KIF18A and KIF18B restored spindle and astral microtubule length, respectively, following BAP1 depletion confirming that it is loss of these proteins that is specifically responsible for these phenotypes. However, as for BRCA1 and MAD2L1, the mechanism through which BAP1 regulates KIF18A and KIF18B expression again remains to be determined. KIF18A is reversibly glycosylated by the *N-*acetylglucosamine (O-GlcNAc) transferase (OGT) enzyme and this may compete with phosphorylation at similar residues influencing the cellular distribution and regulation of KIF18A [73]. Interestingly, OGT localizes to the mitotic spindle during early mitosis, before concentrating at the central spindle and midbody in late mitosis [74]. BAP1 deubiquitinates and stabilizes OGT [75], while CDK1 phosphorylates KIF18A during mitosis at S674/S684. These latter modifications alter the localization of KIF18A and suppress chromosome oscillations, whereas protein phosphatase 1 (PP1) dephosphorylates KIF18A at S674/S684 and stabilizes microtubule attachment at kinetochores [70]. Another interacting partner of BAP1, HCF-1, binds to PP1 and acts as a regulator of PP1 [76]. Hence, BAP1 depletion could affect the stabilization of HCF-1 and OGT thereby impacting on KIF18A expression. There is clearly much more work to be done to explore the BRCA1-independent mechanisms of BAP1 loss that may impact on genome instability. This should include analysing the mitotic phenotypes of BAP1-deficient cells in which BRCA1 expression is restored, as well as testing whether tumour cells undergoing mitotic progression are especially sensitive to loss of BAP1. Furthermore, studies should be undertaken to explore whether BAP1 loss exhibits interactions with other genetic or epigenetic changes that affect mitotic progression in mesothelioma. Finally, it will be important to examine whether the mitotic consequences observed in mesothelioma also occur in other cancer types, such as uveal melanomas, that lack functional BAP1.

The association between BAP1 and chromosome segregation may be clinically relevant. Vinorelbine exhibits useful clinical activity in mesothelioma and BAP1 loss has been correlated with clinical efficacy in the MS01 study [77]. Vinorelbine has been explored in a multicentre randomised phase II trial (VIM, NCT02139904) in which BRCA1 expression was insufficient to predict clinical outcome, in contrast to BAP1 in a retrospective study [4, 77]. Our discovery that BAP1 has additional BRCA1-independent functions in mitotic progression may in part explain these findings. Furthermore, our data that BAP1 inactivation, which is a frequent event in this cancer, can interfere with SAC activity and spindle organization may confer resistance to vinorelbine and this is being explored in the VIM trial. As such, BAP1, which is commonly profiled during routine molecular diagnostic immunohistochemistry, may have potential as a predictive biomarker for microtubule poison drug resistance and thereby underpin chemotherapy stratification.

## Materials & methods

### Cell culture, transfections, and drug treatment

Malignant pleural mesothelioma (MPM) cell lines used in this study were MSTO-211H cells, a biphasic MPM cell line that exhibits a mix of epitheloid and sarcomatoid cell properties with an epithelial to mesenchymal transition phenotype, and NCI-H2452 cells, a MPM cell line with a missense A95D loss-of-function mutation in the UCH domain of BAP1. The A95D mutation in the NCI-H2452 cells was authenticated by Eurofins by PCR single-locus-technology. The MSTO-211H cell line has an integrated shBRCA1 construct that down-regulates the expression of BRCA1 upon doxycycline induction. These cell lines were obtained as described [78]. NCI-H226 parental cells and cells stably expressing BAP1 were a gift from Krishna Kolluri (UCL, London). MEDCL85, MEDCL91 and MEDCL96 are whole exome-sequenced mesothelioma patient-derived primary cell lines obtained from the MEDUSA study [79]. All cell lines were cultured in RPMI 1640 (Gibco) medium supplemented with 10% (v/v) foetal bovine serum (FBS), 50 U/ml of penicillin-streptomycin and, when required, 2 μg/ml puromycin for selection. Cells were maintained in humidified incubators at 37 °C with 5% CO_2,_ except for primary cell lines that were grow with 10% CO_2_. All cell lines were authenticated by STR profiling, tested in-house for mycoplasma by PCR each time they were thawed and grown for no more than ten passages.

Full-length human KIF18A cDNA expression plasmid (Addgene plasmid 23002) was a gift from Linda Wordeman (University of Washington, Seattle), while human KIF18B cDNA expression plasmid was a gift from Julie Welburn (CRUK Cancer Centre, Edinburgh). Plasmids were transfected using Lipofectamine 2000 according to the manufacturer’s instructions. siRNA transfections were done as previously described [80]. siRNA duplexes to BAP1 and BRCA1 (Hs_BAP1_1 FlexiTube siRNA-SI00066696; Hs_BAP1_3 FlexiTube siRNA-SI00066710; Hs_BRCA1_14 FlexiTube siRNA-SI02664361 were obtained from Qiagen and transfected into MSTO-211H or NCI-H2452 cells using Oligofectamine transfection reagent (Invitrogen, UK) according to the manufacturer’s instructions. 72 h after transfection, cells were either fixed for immunocytochemistry or prepared for Western blot or flow cytometry analysis. For proteasome inhibition, cells were treated with 20 µM MG132 (Sigma Aldrich) for 6 h; for monopolar spindle generation, cells were treated with 10 μM of STLC (Sigma Aldrich) for 8 h; mitotic arrest was induced by treatment with 20 µM vinorelbine (Sigma Aldrich) for 24 h.

### Preparation of cell extracts, SDS-PAGE and Western blotting

For preparation of cell extracts, cells were incubated in RIPA lysis buffer (50 mM Tris-HCl pH 8, 150 mM NaCl, 1% v/v Nonidet P-40, 0.1% w/v SDS, 0.5% w/v sodium deoxycholate, 5 mM NaF, 5 mM β-glycerophosphate, 30 µg/ml RNase, 30 µg/ml DNase I, 1x Protease Inhibitor cocktail (PIC), 1 mM PMSF) for 30 min on ice, passed 10 times through a 27 G needle, and supernatants collected by centrifugation at x 13,000 g, 4 °C, 15 min. Protein concentrations of cell lysates were determined using a BCA assay (Pierce). Equalized protein samples were resolved by SDS-PAGE and analysed by Western blotting as previously described [68]. For BRCA1 protein expression 3–8% Tris-acetate gels (Thermofisher) were used. Primary antibodies used were raised against BRCA1 (1:200, rabbit, Santa Cruz Biotechnology, C-20); α-tubulin (1:10000, mouse, Thermofisher, PA5-29444); BAP1 (1:500, mouse, Santa Cruz Biotechnology, C-4); KIF18A (1:200, rabbit, Bethyl Laboratories, A301-080A); KIF18B (1:200, rabbit, Bethyl Laboratories, A303-982A); MAD2L1 (1:500, rabbit, Thermofisher, PA5-21594); BUBR1 (1:500, mouse, Abcam, ab28193). Secondary antibodies used were anti-rabbit or anti-mouse horseradish peroxidase (HRP)-labelled IgGs (1:1000; Sigma). Western blots were developed using an enhanced chemiluminescence (ECL) detection kit (Pierce), except for BRCA1 when blots were developed using WesternBright ECL (Advansta).

### Immunofluorescence microscopy

Cells grown on acid-etched coverslips were fixed and permeabilised using ice-cold methanol for 20 min or overnight. Coverslips were incubated with blocking buffer (1% BSA/PBS) for 1 h and washed 3x with PBS for 5 min each. All primary antibody dilutions were done in 3% BSA/PBS for 2 h. Primary antibodies used were against GFP (0.5 µg/ml, rabbit; ab6556); α-tubulin (1:500, rabbit, Thermofisher, PA5-29444); α-tubulin (1:250, mouse, Thermofisher, DM1A); γ-tubulin (1:500, mouse, Abcam, ab27074); γ-tubulin (1:500, rabbit, Abcam, ab11321); CDK5RAP2 (1:500, rabbit, Bethyl Laboratories, IHC-00063); C-Nap1 (1:500, rabbit, Proteintech, 14498-1-AP); BUBR1 (1:200, mouse, Abcam, ab28193). Coverslips were washed in PBS and incubated with secondary antibodies in PBS supplemented with 3% BSA for 1 h. Secondary antibodies used were Alexa Fluor 488 and 594 goat anti-rabbit and goat anti-mouse IgGs (10 µg/ml, Invitrogen). In addition, 0.8 µg/ml Hoechst 33258 was used to stain DNA. Coverslips were mounted in 80% glycerol, 3% n-propylgallate in PBS mounting medium. Fluorescence images were obtained using a Leica TCS SP5 laser scanning confocal microscope equipped with a Leica DMI 6000B inverted microscope using a Plan Apo 63x oil objective (NA 1.4). Images were captured and processed using Leica LAS AF software or ImageJ. Deconvolution of 3D image stacks was performed using Huygens Essential software (Scientific Volume Imaging).

### Image quantifications

All image quantifications except BUBR1 intensity measurements were performed using Imaris 3D software. Quantification of centrosome volume of cells in interphase and mitosis based on γ-tubulin and CDK5RAP2 staining were done using surface selection tool to render solid surfaces to measure centrosome volume. Spindle length was measured as pole-to-pole distance using slice selection. Microtubule length in monopolar spindles was measured from the middle of the monopolar aster to the tip of the microtubule. KIF18A intensity and aster volume was measured using object counter. Aster volumes were measured as volume around the microtubules radiating from the centrosome. BUBR1 intensity was quantified using ImageJ. All measurements are based on the mean intensity and volume was calculated in μm^3^. Centrosome numbers and multipolar spindles were counted manually.

### Immunohistochemistry

Tissue samples were obtained from patients enrolled in the MEDUSA cohort with appropriate ethical and governance approval as previously described [79]. Fresh sections from patient tissues were cut at a thickness of 4 μm. Slides were incubated at 60 °C for an hour to excess wax removal. Slides were deparaffinized and pre-treated in Dako PT Link (pre-treatment) with target retrieval solution, high or low pH for an hour. Slides were transferred to Dakolink 48 autostainer and queued using barcodes with their respective antibodies for four hours. Images were taken at 40x magnification on a Hamamatsu Nanozoomer digital slide scanner. Antibodies were used against BRCA1 (Abcam ab213929) and BAP1 (Santa Cruz sc28383). Scoring was carried out by a pathologist with orthogonal validation using image process by Qupath and BAP1- and BRCA1-positive tumours defined as those demonstrating >10% of cells with nuclear stain.

### Statistical analysis

One-sided Student’s t-test was used to determine the statistical differences among the analysed groups. Results are presented as the mean of three independent experiments, unless otherwise stated. Error bars represent standard deviation of the mean (S.D., *n* = 3). Statistical analysis was performed using Prism 7.0 software.

## Supplementary information

Supplementary Material

## Data Availability

The datasets generated and analysed during the current study are either included in this published article (and its supplementary information files) or available from the corresponding author on reasonable request.
