# Supplementary material for: BAP1 loss induces mitotic defects in mesothelioma cells through BRCA1-dependent and independent mechanisms
Source: Oncogene. 2022 Dec 22;42(8):572–85. doi: 10.1038/s41388-022-02577-3 (PMC9937923; doi:10.1038/s41388-022-02577-3)
Supplement: Supplementary file 1 — Supplementary Material [file 41388_2022_2577_MOESM1_ESM.pdf]

Supplementary Material

BAP1 loss induces mitotic defects in mesothelioma cells through BRCA1-dependent and independent mechanisms

Anita Singh[1,2], Sara Busacca[2], Aarti Gaba[2], Michael Sheaff[3], Charlotte Poile[2], Apostolos Nakas[4], Joanna Dzialo[2], Aleksandra Bzura[2], Alan G. Dawson[2,4], Dean A. Fennell[2,4] and Andrew M. Fry[1,5]

[1]Department of Molecular and Cell Biology, University of Leicester, Lancaster Road, Leicester LE1 9HN, U.K.
[2]Leicester Cancer Research Centre, Department of Genetics and Genome Biology, University of Leicester, Robert Kilpatrick Clinical Sciences Building, Leicester LE2 7LX, U.K.
[3]Department of Histopathology, Barts Health NHS Trust, Queen Mary University of London, The Royal London Hospital, London E1 2ES, U.K.
[4]University Hospitals of Leicester NHS Trust, Glenfield Hospital, Leicester LE3 9QP, U.K.

SUPPLEMENTARY FIGURE LEGENDS

Figure S1. Depletion of BAP1 or BRCA1 does not alter cell cycle progression in mesothelioma cell lines

A. MSTO-211H (left) and NCI-H2452 (right) cells were either mock-depleted or depleted with siRNAs against BRCA1 or BAP1 for 72 h, or by induction of an shBRCA1 sequence for 48 h as indicated. Cells were then fixed, stained with propidium iodide and analysed by flow cytometry. The % cells in G2/M are indicated. Data are expressed as means ±S.D. (n=3). **B**. Representative flow cytometry profiles from which % G2/M populations shown in A are taken.

Figure S2. BAP1 depletion leads to mitotic progression defects and loss of SAC integrity

A. MSTO-211H and NCI-H2452 were either mock-depleted or depleted of BAP1 or BRCA1 as described in Fig. S1A. Cells were stained with antibodies against CENP-A (red); DNA was stained with Hoechst 33258 (blue). Cells in metaphase are indicated and illustrate examples of misaligned chromosomes as quantified in Figure 2B. **B.** Cells treated as in A were stained with antibodies against CENP-A (red); DNA was stained with Hoechst 33258 (white). Examples of interphase cells with micronuclei (left) and multinucleated cells (right) are shown as quantified in Figure 2C & D. **C**. MSTO-211H cells depleted of BAP1 or BRCA as described in A and then either untreated or treated with vinorelbine for 24 hours were analysed by Western blot with antibodies against BUBR1 and α-tubulin. **D**. Cells depleted of BAP1 or BRCA1 as in A were then treated with vinorelbine for 24 hours before being fixed and stained with antibodies against BUBR1 (green); DNA was stained with Hoechst 33258 (red). Scale bars in A, B and D, 5 µm.

Figure S3. Loss of BAP1 regulates spindle organization through KIF18 proteins

A. MSTO-211H cells were mock-depleted or depleted of BAP1 for 48 h prior to transfection of GFP-KIF18B for 24 h. Cells were then analysed by immunofluorescence microscopy with antibodies against α-tubulin (green). Merge images include DNA stained with Hoechst 33258 (blue). Magnified views of astral microtubules are shown and represent examples of images from which astral microtubule volumes shown in in Figure 6E were quantified. Scale bar, 5 µm. **B**. Spindle lengths were calculated based on pole-to-pole distances from MSTO-211H cells treated as in A. Data are expressed as means ±S.D. (n=3). **C**. Lysates were prepared from parental NCI-H226 cells or NCI-H226 cells stably expressing wild-type BAP1 (+BAP1) and Western blotted with antibodies against BAP1, KIF18A or α-tubulin.

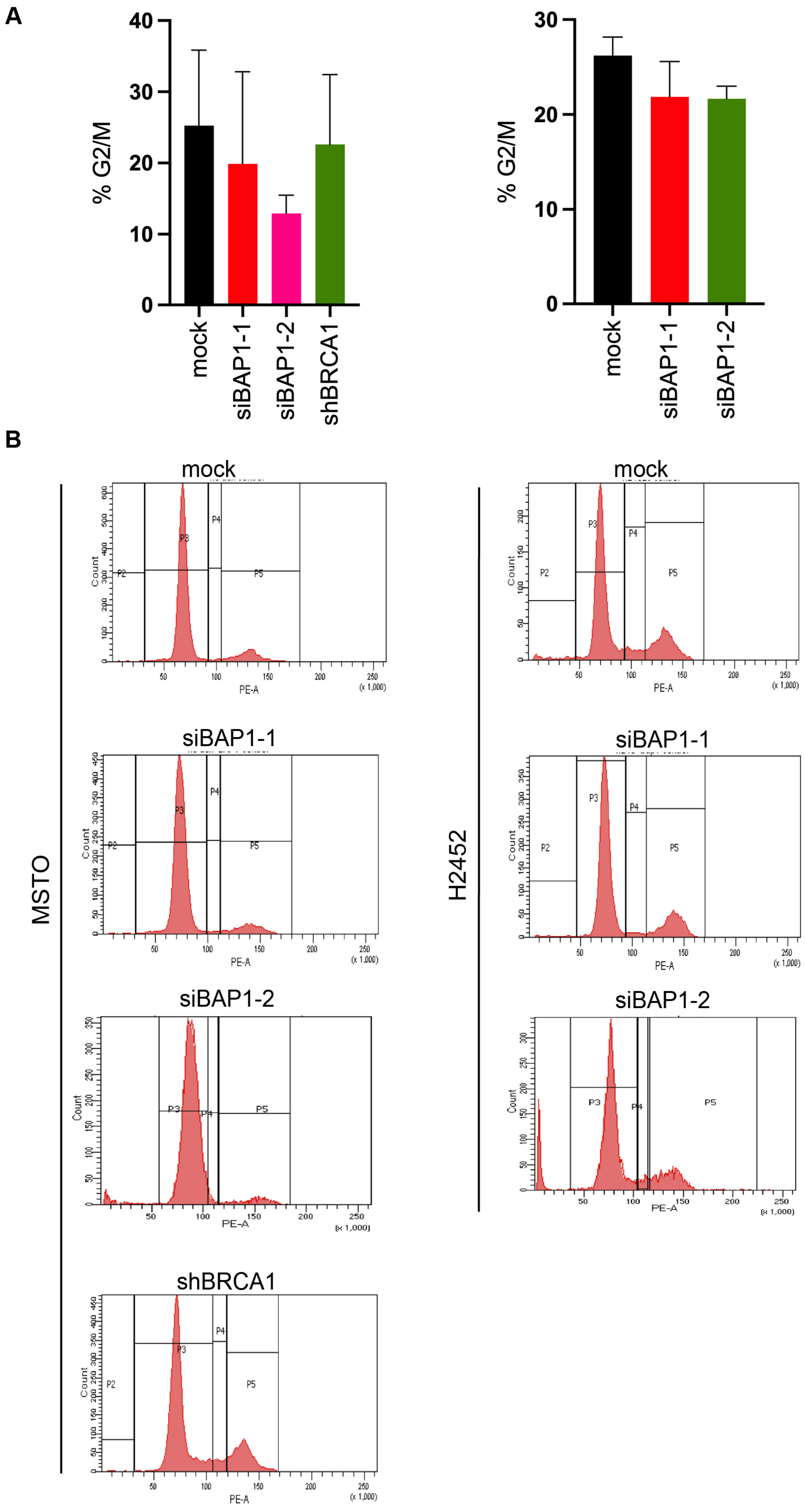
A
% G2/M
40
30
20
10
0
mock
siBAP1-1
siBAP1-2
shBRCA1
% G2/M
30
20
10
0
mock
siBAP1-1
siBAP1-2
B
MSTO
H2452
mock
siBAP1-1
siBAP1-2
shBRCA1
mock
siBAP1-1
siBAP1-2
P2
P3
P4
P5
Count
PE-A
(x 1,000)

Singh et al. Figure S2

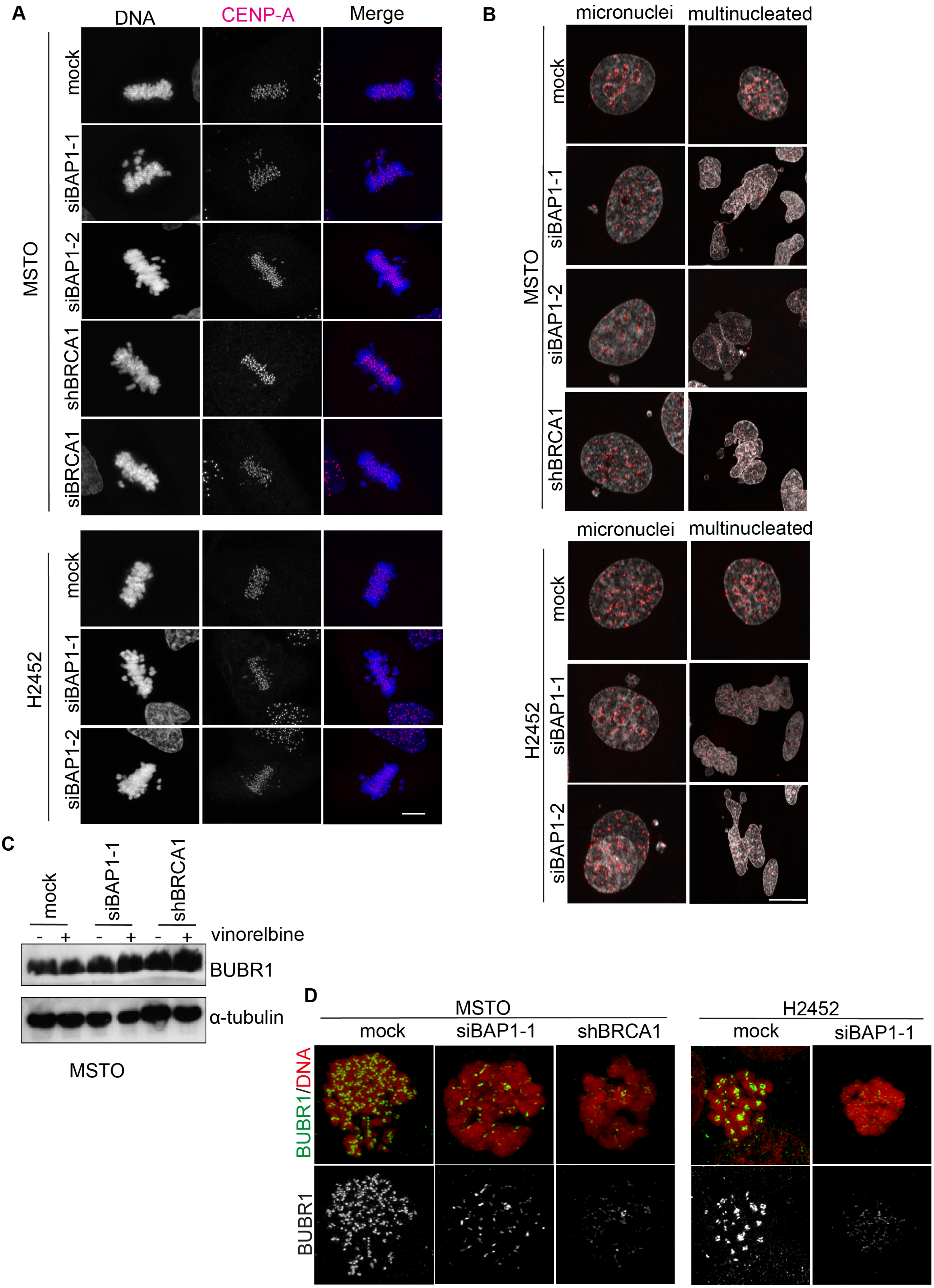

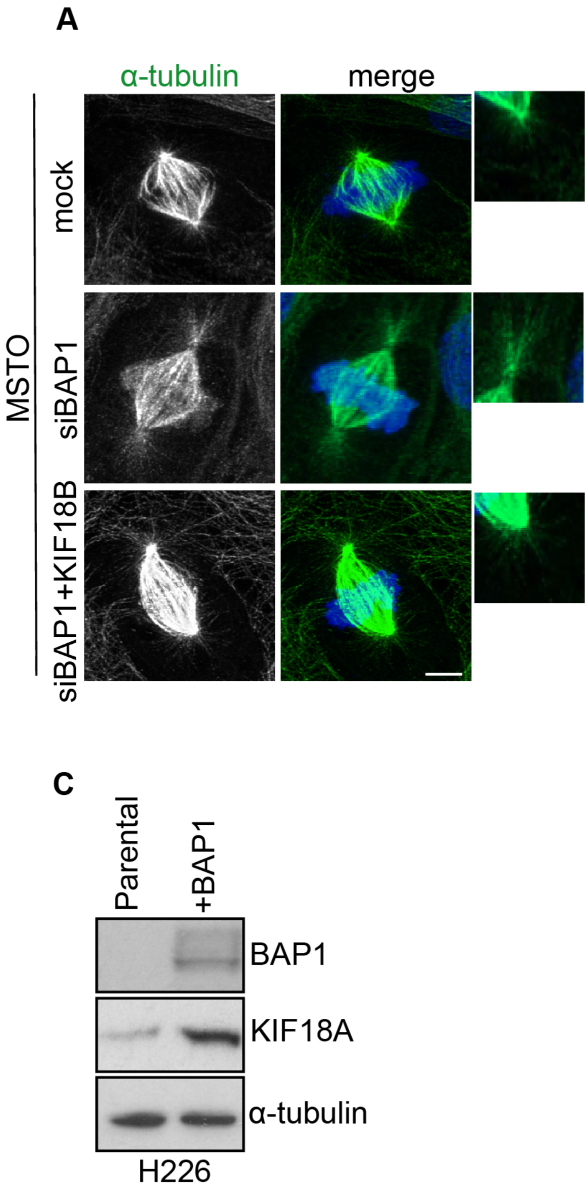
A
α-tubulin
merge
MSTO
mock
siBAP1
siBAP1+KIF18B
B
****
spindle length (μm)
20
15
10
5
0
mock
siBAP1-1
siBAP1-1 +KIF18B
C
Parental
+BAP1
BAP1
KIF18A
α-tubulin
H226
